# Diet and irradiation effects on the bacterial community composition and structure in the gut of domesticated teneral and mature Queensland fruit fly, *Bactrocera tryoni* (Diptera: Tephritidae)

**DOI:** 10.1186/s12866-019-1649-6

**Published:** 2019-12-24

**Authors:** Deane N. Woruba, Jennifer L. Morrow, Olivia L. Reynolds, Toni A. Chapman, Damian P. Collins, Markus Riegler

**Affiliations:** 1 Plant Biosecurity Cooperative Research Centre, LPO, Box 5012, Bruce, ACT 2617 Australia; 2 Hawkesbury Institute for the Environment, Western Sydney University, Locked Bag 1797, Penrith, NSW 2751 Australia; 3 Biosecurity and Food Safety, NSW Department of Primary Industries, Elizabeth Macarthur Agricultural Institute, Private Bag 4008, Narellan, NSW 2567 Australia; 4 Graham Centre for Agricultural Innovation (an alliance between Charles Sturt University and NSW DPI), Locked Bag 588, Wagga Wagga, NSW 2678 Australia; 5 cesar Pty Ltd, 293 Royal Parade, Parkville, Victoria 3052 Australia; 6 Biometrics Unit, NSW Department of Primary Industries, Elizabeth Macarthur Agricultural Institute, Private Bag 4008, Narellan, NSW 2567 Australia

**Keywords:** Sterile insect technique, microbiome, 16S rRNA gene, Enterobacteriaceae, Acetobacteraceae, Asaia sp.

## Abstract

**Background:**

Mass-rearing, domestication and gamma irradiation of tephritid fruit flies used in sterile insect technique (SIT) programmes can negatively impact fly quality and performance. Symbiotic bacteria supplied as probiotics to mass-reared fruit flies may help to overcome some of these issues. However, the effects of tephritid ontogeny, sex, diet and irradiation on their microbiota are not well known.

**Results:**

We have used next-generation sequencing to characterise the bacterial community composition and structure within Queensland fruit fly, *Bactrocera tryoni* (Froggatt)*,* by generating 16S rRNA gene amplicon libraries derived from the guts of 58 individual teneral and mature, female and male, sterile and fertile adult flies reared on artificial larval diets in a laboratory or mass-rearing environment, and fed either a full adult diet (i.e. sugar and yeast hydrolysate) or a sugar only adult diet. Overall, the amplicon sequence read volume in tenerals was low and smaller than in mature adult flies. Operational taxonomic units (OTUs), belonging to the families Enterobacteriaceae (8 OTUs) and Acetobacteraceae (1 OTU) were most prevalent. Enterobacteriaceae dominated laboratory-reared tenerals from a colony fed a carrot-based larval diet, while Acetobacteraceae dominated mass-reared tenerals from a production facility colony fed a lucerne chaff based larval diet. As adult flies matured, Enterobacteriaceae became dominant irrespective of larval origin. The inclusion of yeast in the adult diet strengthened this shift away from Acetobacteraceae towards Enterobacteriaceae. Interestingly, irradiation increased 16S rRNA gene sequence read volume.

**Conclusions:**

Our findings suggest that bacterial populations in fruit flies experience significant bottlenecks during metamorphosis. Gut bacteria in teneral flies were less abundant and less diverse, and impacted by colony origin. In contrast, mature adult flies had selectively increased abundances for some gut bacteria, or acquired these bacteria from the adult diet and environment. Furthermore, irradiation augmented bacterial abundance in mature flies. This implies that either some gut bacteria were compensating for damage caused by irradiation or irradiated flies had lost their ability to regulate bacterial load. Our findings suggest that the adult stage prior to sexual maturity may be ideal to target for probiotic manipulation of fly microbiota to increase fly performance in SIT programmes.

## Background

In the quest to understand the association between bacteria and their insect hosts, one of the first associations studied was a tephritid fruit fly pest, the olive fly, *Bactrocera oleae* (Rossi) (Diptera: Tephritidae) and its gut microbiota [1]. Over the past decade, there has been increasing interest in symbiosis of bacteria with tephritids, particularly the potential manipulation of this association for pest management [2, 3]. One such prospect involves the use, or manipulation of microbial symbionts as part of the sterile insect technique (SIT) [4].

SIT involves mass rearing (leading to domestication [5]) and the release of irradiated (sterile) individuals of the target pest species into wild pest populations in the field [6]. The success of SIT relies upon sterile males locating and successfully copulating with field females, resulting in embryonic mortality and a decline of the pest population. However, released sterile tephritid males are less competitive than their field male counterparts due to the processes of mass-rearing and exposure to ionizing radiation [7].

Tephritids are holometabolous insects with different nutritional environments and requirements during their developmental stages [8]. To attain sexual maturity and achieve good sexual performance certain nutrients are relevant, particularly at the adult stage [9]. For example, yeast, as a protein source, is known to affect adult male and female tephritid fitness and performance differently during the development [10]. Fruit flies harbour symbiotic bacteria in their gut and research suggests that they are involved in the fly’s nutritional status. As environmental factors are known to shape the composition and structure of bacterial communities in tephritids [11], tephritid development may also impact their gut microbiome and therefore condition how resources are used. Furthermore, diets and exposure to irradiation are known to affect the performance of mass-reared adult tephritids [12]. Although it is known that exposure to irradiation damages the tephritid gut [13], little is known about how this affects the gut microbiome. In this sense, a supplementation of symbiotic bacteria to mass-reared irradiated tephritids is expected to improve their performance [14, 15]. Therefore, an improved understanding of the gut bacterial communities, and how they are impacted by insect development and environmental factors (such as diets and irradiation) may lead to the identification of beneficial symbiotic gut bacteria and how these may be promoted in flies, e.g. through probiotic supplementation.

In Australia, SIT is used in an integrated approach to control the serious horticultural pest, Queensland fruit fly, *Bactrocera tryoni* (Froggatt) (Diptera: Tephritidae) [16, 17]. The aim of the present study was to investigate the bacterial community composition and structure within the gut of domesticated populations of *B. tryoni* flies and determine the effects of colony origin, adult developmental stage, sex, adult diets, rearing environment, and exposure to gamma irradiation on gut microbiota. We hypothesised that diet and irradiation affect the gut microbiome. We used 16S rRNA gene amplicon next-generation sequencing (NGS) to characterise the gut bacterial communities of teneral (immature) and mature adult *B. tryoni*, irradiated and unirradiated, maintained on varying adult diets in order to understand the bacterial population dynamics across adult development and to identify an optimal time point for adult probiotic supplementation to enhance adult fruit fly performance for SIT.

## Methods

### Treatment of teneral and mature adults

The flies for the characterisation of the bacterial communities were sampled from two colonies of *B. tryoni* in late January 2015. These two colonies were originally sourced from two different field-collected lines and then independently maintained at two rearing facilities that used larval diets comprising different bulking agents. The first *B. tryoni* colony was from the Fruit Fly Production Facility (FFPF) of the Elizabeth Macarthur Agricultural Institute (EMAI), NSW Department of Primary Industries (NSW DPI) in Menangle, New South Wales (NSW). This colony was maintained for use in the Queensland fruit fly SIT program and was sourced from a line derived from *B. tryoni* infested fruits collected in the NSW Central Coast region in 2013 and established at the NSW DPI’s Central Coast Primary Industries Centre (CCPIC), in Ourimbah, NSW. At the FFPF, mass-reared individuals (> 5 million/week; 5000 larvae per litre larval diet [personal communication S Balagawi]) of this 2 year-old colony were reared on standard fruit fly larval growth medium using lucerne chaff as the bulking agent, torula yeast, white cane sugar, water, citric acid, sodium benzoate and methyl paraben [18].

The second *B. tryoni* colony (BtGWS in [11]) was from the laboratory of the Hawkesbury Institute for the Environment (HIE), Western Sydney University, Richmond, NSW. This colony was maintained for research purposes and originated from a CCPIC line established from infested fruits collected in the field in the NSW Central West region in 2009. At HIE, laboratory-reared individuals (< 500/cohort; approximately 3000 larvae per litre larval diet) of this 6 year-old laboratory colony [11] were reared on a larval diet consisting of dehydrated ground carrot as the bulking agent, torula yeast, water, hydrochloric acid and methyl paraben [19]. A key compositional difference between the two larval diets was the bulking agents (lucerne chaff versus ground carrot) that have minimal nutritional function, but rather provide a matrix to allow aeration and heat dissipation as the larvae feed and develop within the diet.

To cause sterility, half of the late-stage FFPF pupae were irradiated in a ^60^Co in-ground gamma Technology Research Irradiator at the Australian Nuclear Science and Technology Organisation (ANSTO) in Lucas Heights, NSW, at the current recommended dose of 60–65 Gy and a dose rate of approximately 6 Gy min^− 1^, while a second group of FFPF pupae were not irradiated and remained fertile. All pupae of the HIE cohort were fertile, i.e. unirradiated.

Adult flies were sampled from 18 experimental treatment groups based on adult developmental stage (teneral or mature adults), larval rearing environment (FFPF and HIE populations reared on different larval diets), irradiation status (irradiated or unirradiated), sex (male or female) and adult diet (sugar only, or full diet, i.e. 3:1 ratio of white sugar and yeast hydrolysate) (Table 1). In preparation for this, approximately 100 pupae from each of the experimental populations were set up in Petri dishes in separate 30 cm × 30 cm × 30 cm mesh covered cages (BugDorm, Taiwan) in a controlled glasshouse chamber at HIE at 25 ± 3 °C, 65 ± 15% RH and a 10:14 h light: dark photoperiod. The cages were monitored three times daily and flies sampled as teneral and mature adults. Tenerals were not provided with water or food and were sampled between 6 and 12 h post eclosion (tenerals less than 6 h old were not used as their digestive systems were soft and disintegrated when dissected). Captive adult *B. tryoni* reach maturity by 10 days [19, 20]. Therefore, mature adults were sampled at 14 days, and were provided with water and either a full adult diet (sugar and yeast hydrolysate [3:1]) or a sugar only adult diet from eclosion. All adult diets were provided as 2% agar in a Petri dish [21]. The adult diets were replaced every second day.

**Table 1:** *Bactrocera tryoni* experimental treatment groups

Treatment group	Life stage	Colony origin	Larval diet bulking agent	Adult diet	Irradiation	Sex
ELNIF	Teneral	EMAI-FFPF	Lucerne chaff	Nil	Irradiated	Female
ELNIM	Teneral	EMAI-FFPF	Lucerne chaff	Nil	Irradiated	Male
ELNUF	Teneral	EMAI-FFPF	Lucerne chaff	Nil	Unirradiated	Female
ELNUM	Teneral	EMAI-FFPF	Lucerne chaff	Nil	Unirradiated	Male
HCNUF	Teneral	HIE	Carrot	Nil	Unirradiated	Female
HCNUM	Teneral	HIE	Carrot	Nil	Unirradiated	Male
ELSIF	Mature	EMAI-FFPF	Lucerne chaff	Sugar only	Irradiated	Female
ELYIF	Mature	EMAI-FFPF	Lucerne chaff	Full diet	Irradiated	Female
ELSIM	Mature	EMAI-FFPF	Lucerne chaff	Sugar only	Irradiated	Male
ELYIM	Mature	EMAI-FFPF	Lucerne chaff	Full diet	Irradiated	Male
ELSUF	Mature	EMAI-FFPF	Lucerne chaff	Sugar only	Unirradiated	Female
ELYUF	Mature	EMAI-FFPF	Lucerne chaff	Full diet	Unirradiated	Female
ELSUM	Mature	EMAI-FFPF	Lucerne chaff	Sugar only	Unirradiated	Male
ELYUM	Mature	EMAI-FFPF	Lucerne chaff	Full diet	Unirradiated	Male
HCSUF	Mature	HIE	Carrot	Sugar only	Unirradiated	Female
HCYUF	Mature	HIE	Carrot	Full diet	Unirradiated	Female
HCSUM	Mature	HIE	Carrot	Sugar only	Unirradiated	Male
HCYUM	Mature	HIE	Carrot	Full diet	Unirradiated	Male

Treatment group abbreviations represent treatments for individual samples with the first letter indicating the colony origin of either EMAI-FFPF (E) or HIE (H) from which the pupae were collected, the second letter indicating the larval diets of either carrot (C) or lucerne chaff (L), the third letter identifies the adult diet of either a full adult diet consisting of yeast hydrolysate and sugar (3:1) (Y), sugar only (S) or nil (N) as in the case of tenerals who were not fed, the fourth letter indicates if the pupae were irradiated (I) or unirradiated (U), the fifth letter denotes the sex, either male (M) or female (F). Adult diets were provided in a 1% agar matrix)

### Gut dissection

At least three samples of *B. tryoni* from each of the 18 experimental treatment groups (Table 1) were selected for gut dissections. Insects were placed in 250 mL specimen jars and, within 30 min of sampling, were anaesthetised with carbon dioxide for 1 min. The insects were then surface sterilised by sequentially immersing for 1 min in each of 70% ethanol, sterile distilled water, 0.05% sodium hypochlorite and lastly sterile distilled water, before individuals were placed on a sterile concave glass slide that had been surface treated by wiping with 70% ethanol and 0.05% sodium hypochlorite. The glass slide was placed on top of ice in a plastic Petri dish, which was then viewed under a stereomicroscope. Two pipette drops of sterile phosphate-buffered saline (PBS) were placed on top of the insect before dissection with sterile forceps. The dissection involved firstly removing the wings, the legs and the exoskeleton after softening by immersion in PBS for 1 min. The intact gut of the insects was then gently removed and placed in a clean 1.5 mL microcentrifuge tube and immediately transferred to a freezer (− 20 °C) for a maximum of 1 h. Afterwards, samples were stored at − 80 °C until required.

### DNA extraction, library preparation and 16S rRNA gene amplicon sequencing

DNA from each of 58 individual gut samples stored at − 80 °C was extracted using the QIAmp DNA mini kit (Qiagen), including RNase treatment, and eluted in 50 μL nuclease-free water. DNA integrity was examined by gel electrophoresis. The DNA solutions were reduced to a volume between 15 and 20 μL using a vacuum concentrator. DNA concentration and purity were assessed using Qubit 2.0 Fluorometry and Nanodrop spectrophotometry. Each genomic DNA sample was also PCR amplified using the eubacterial 16S rRNA gene primers 63F and 1227R, and insect mitochondrial COI with primers Pat and Dick, as described in Morrow et al. [22], to ensure the DNA did not contain inhibitors that would interfere with amplification.

The DNA samples were then submitted for high-throughput sequencing at the HIE Next-Generation Sequencing Facility for 16S rRNA gene amplification of 7 ng DNA using primers 341F – 5′ CCTACGGGNGGCWGCAG 3′ and 805R – 5′ GACTACHVGGGTATCTAATCC 3′, which span the variable V3 and V4 regions of the 16S rRNA gene producing a fragment of approximately 464 bp. Library preparation for 58 samples was performed with the Nextera XT kit, and sequencing of 2 × 300 bp paired ends was performed on a 384-multiplexed Illumina MiSeq run.

### Sequence analyses

The data was analysed using the open-source bioinformatics pipeline QIIME [23]. The raw data of the 58 libraries received in fastq format were examined using FastQC v0.11.5 [24], which showed that trimming of at least 10 bp from the 3′ ends of R1 reads and 90 bp from R2 reads would improve the quality of the merged sequences. Therefore, the reads were trimmed using the *trimfq* command of seqtk [25], removing the primer and the final 10 bp (−b 17 –e 10) from the forward (R1) reads, as well as the primer and final 90 bp from the reverse (R2) reads (−b 21 –e 90). FLASH v1.2.11 [26] was used to join the trimmed, paired reads into single sequences with a minimum overlap of 10 bp.

The operational taxonomic units (OTUs) were assigned using the *pick_open_reference_otus.py* command which also removes singletons. Chimeric sequences were detected and removed using ChimeraSlayer [27].

After singleton and chimera removal, the number of sequence reads per library and alpha diversity indices were compared by pairwise ANOVA and plotted by using *base R* commands in R [28]. Due to the significant difference in sequence read numbers obtained per library, following quality control, the data were split into two groups defined as teneral adults and mature adults, and then the sequences for each group were normalised to the lowest number of sequences found in each group using the command *single_rarefaction.py*. The rarefaction curves to assess coverage were created by the *rarecurve* command of the Vegan package [29] in R.

Beta diversity across the samples was analysed by the phylogenetic distance-based measurement, UniFrac and the abundance distance-based measurement, Bray-Curtis. The distance matrix values for unweighted UniFrac (presence and absence of taxa), weighted UniFrac (presence, absence and abundance of taxa) and Bray-Curtis (compositional dissimilarity based on counts) for the samples were calculated in QIIME. Then, the distance matrices were imported into R for statistical analysis of treatment effects and plotting of the principal component analysis (PCoA) and relative abundance. The ellipses in the PCoA plots were created using the *ordiellipse* command of the Vegan package and the heatmap plots were created using the *levelplot* command of the Lattice package [30] in R.

## Results

### Sequence read analyses

A total of 58 libraries from 19 teneral and 39 mature adult *B. tryoni* were high-throughput amplicon sequenced for approximately 460 bp of their bacterial 16S rRNA gene with the primers 341F and 805R. This generated 2,453,686 raw sequence reads (Additional file 1 Table S1). After filtering, 1,088,483 (44.4%) sequences remained and this large reduction in sequence read numbers was likely due to the reads being of low quality at the 3′ ends, which affects the number of read pairs that are merged into a complete sequence fragment, both by reducing the amount of overlap found in reads producing a larger merged sequence (i.e. ~ 426 bp), or by having too much overlap in smaller sized sequences (i.e. ~ 403 bp) and mismatches preventing the reads from being merged. Standardised trimming parameters were applied across all samples in order to minimise bias in merging the paired reads. Clustering at 97% identity across all samples, produced 727 OTUs (Additional file 2, Additional file 3: Table S4). Following chimera removal there were 324 OTUs across the entire dataset, including 44 OTUs in tenerals and 309 OTUs in mature adults, and sequences were reduced to 1,018,739 (41.5%) ranging from 11 to 19,606 in tenerals and 7850 to 57,800 in mature adults.

**Fig. 1:**
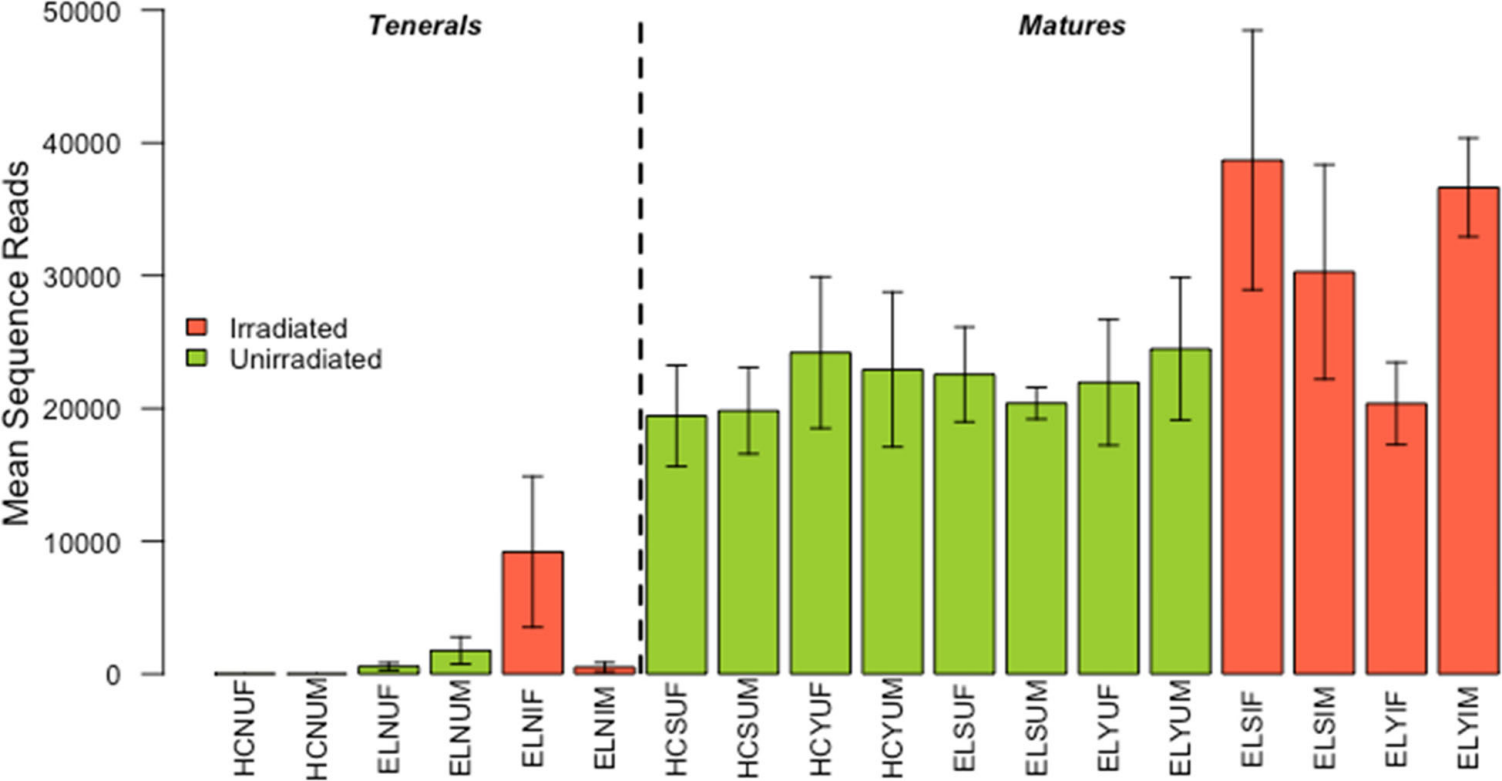
Mean of counts of 16S rRNA gene reads by treatment groups. Letter codes are as per Table 1

The comparative number of 16S rRNA gene sequence reads across libraries can be used as an indicator of the relative bacterial load across samples. The total sequence reads, or bacterial loads were higher in mature adults (*x̅* = 25,190.36 ± 1674.84 SE) than in tenerals (*x̅* = 1911.32 ± 1076.351 SE) (*F*_1,57_ = 85.15, *p* < 0.001; Fig. 1 and Additional file 1 Table S2). The colony origin affected sequence reads in tenerals (*F*_1,12_ = 5.23, *p* < 0.05) where FFPF tenerals (*x̅* = 1167.00 ± 544.80 SE) had more reads than HIE tenerals (*x̅* = 22.14 ± 4.01 SE). The irradiation of pupae also resulted in a higher count of sequence reads in mature adults (*F*_1,25_ = 4.89, *p* = < 0.05) with irradiated matures (*x̅* = 31,403.08 ± 3676.84 SE) having more sequence reads than unirradiated matures (*x̅* = 22,367.69 ± 1780.03 SE). The other parameters of sex and adult diet (for mature adults only), had no discernible impact on bacterial sequence read count (Additional file 1 Table S2).

### Alpha diversity

**Fig. 2:**
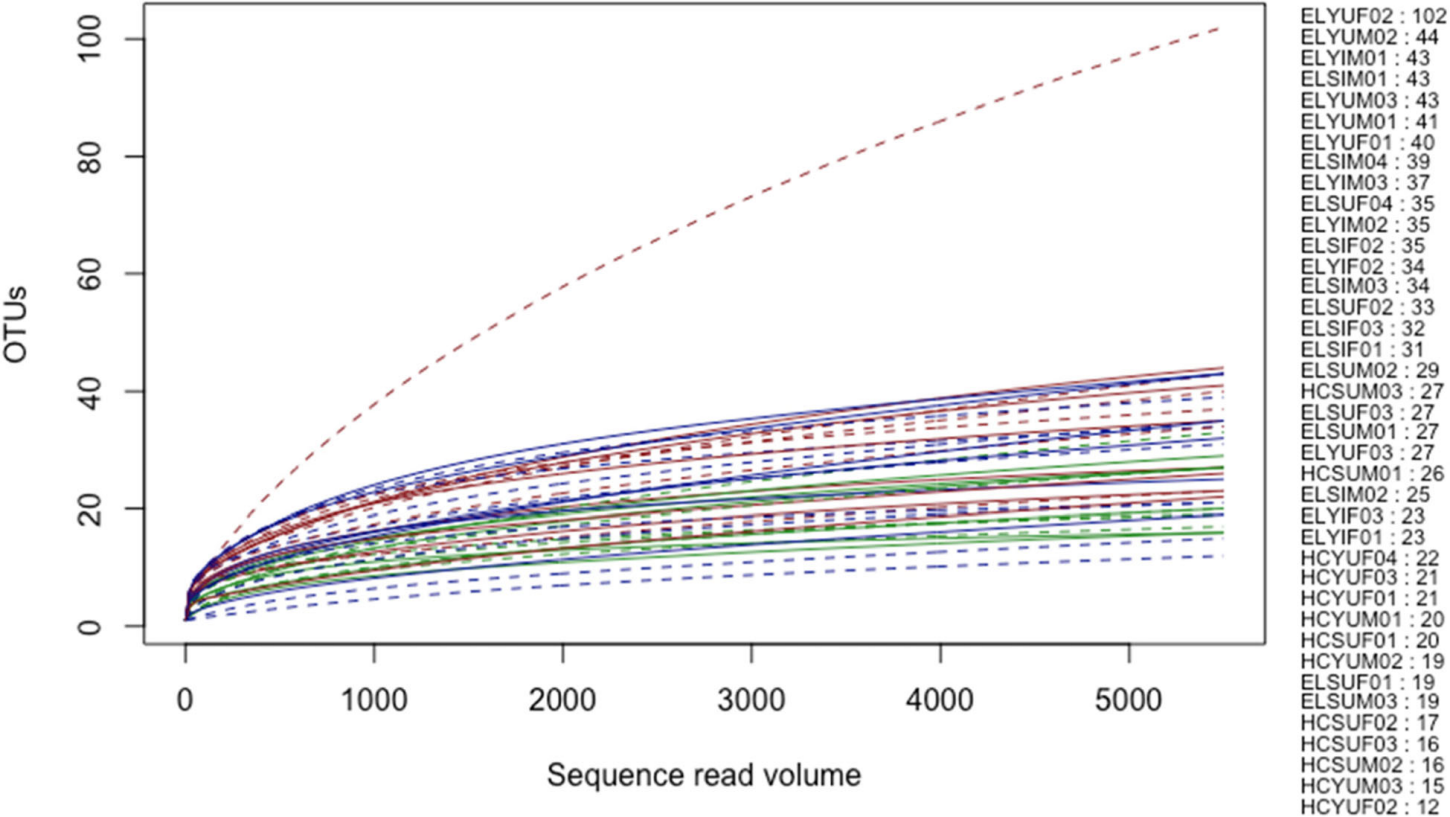
Rarefaction curves for mature *Bactrocera tryoni*. Figures to the right of the graph indicate the order of lines as sorted by number of OTUs. Sample name letter codes are as per Table 1

**Fig. 3:**
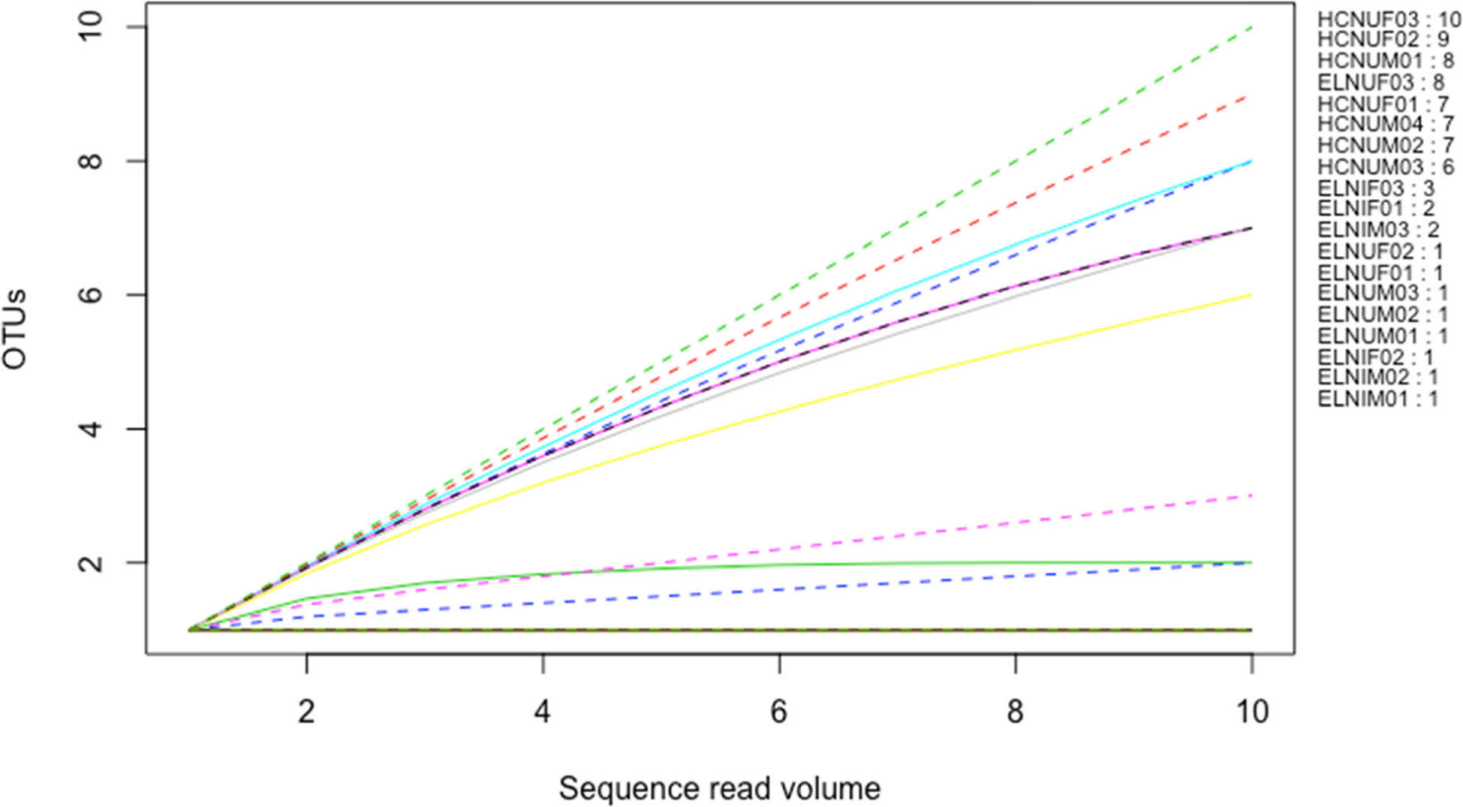
Rarefaction curves for teneral *Bactrocera tryoni*. Figures to the right of the graph indicate the order of lines as sorted by number of OTUs. Sample name letter codes are as per Table 1

Rarefaction curve (Fig. 2) and Good’s coverage (Additional file 1 Table S1) indicated that microbial communities of the mature adults were well captured by the sequencing coverage. The reads from the mature fly samples were rarefied to 5500 and were represented by 309 observed OTUs. The most OTU diverse mature sample was one unirradiated female, kept on a full adult diet (containing yeast hydrolysate and sugar), originating from a FFPF pupa (ELYUF02) that contained 102 OTUs. The rest of the mature samples were much less diverse and contained between 12 and 44 OTUs. The sequences from the tenerals clustered into 44 OTUs following rarefaction to 10 sequences per sample (Fig. 3), but only nine out of 19 samples achieved adequate sequence coverage at this low value.

Colony origin significantly influenced the number of OTUs in both mature (*F*_1,25_ = 9.055, *p* < 0.01) and teneral flies (*F*_1,12_ = 20.93, *p* < 0.001) (Additional file 1 Table S3). Mature adult flies derived from FFPF pupae had more OTUs (*x̅* = 37.39 ± 5.86 SE) than mature flies derived from HIE pupae (*x̅* = 19.39 ± 1.18 SE). Contrary to the mature adults, HIE tenerals (*x̅* = 7.71 ± 0.52 SE) were more OTU diverse than FFPF tenerals (*x̅* = 2.17 ± 1.17 SE). Sex, irradiation, and adult diets were observed to not affect the number of OTUs in adult flies.

### Beta diversity

**Fig. 4:**
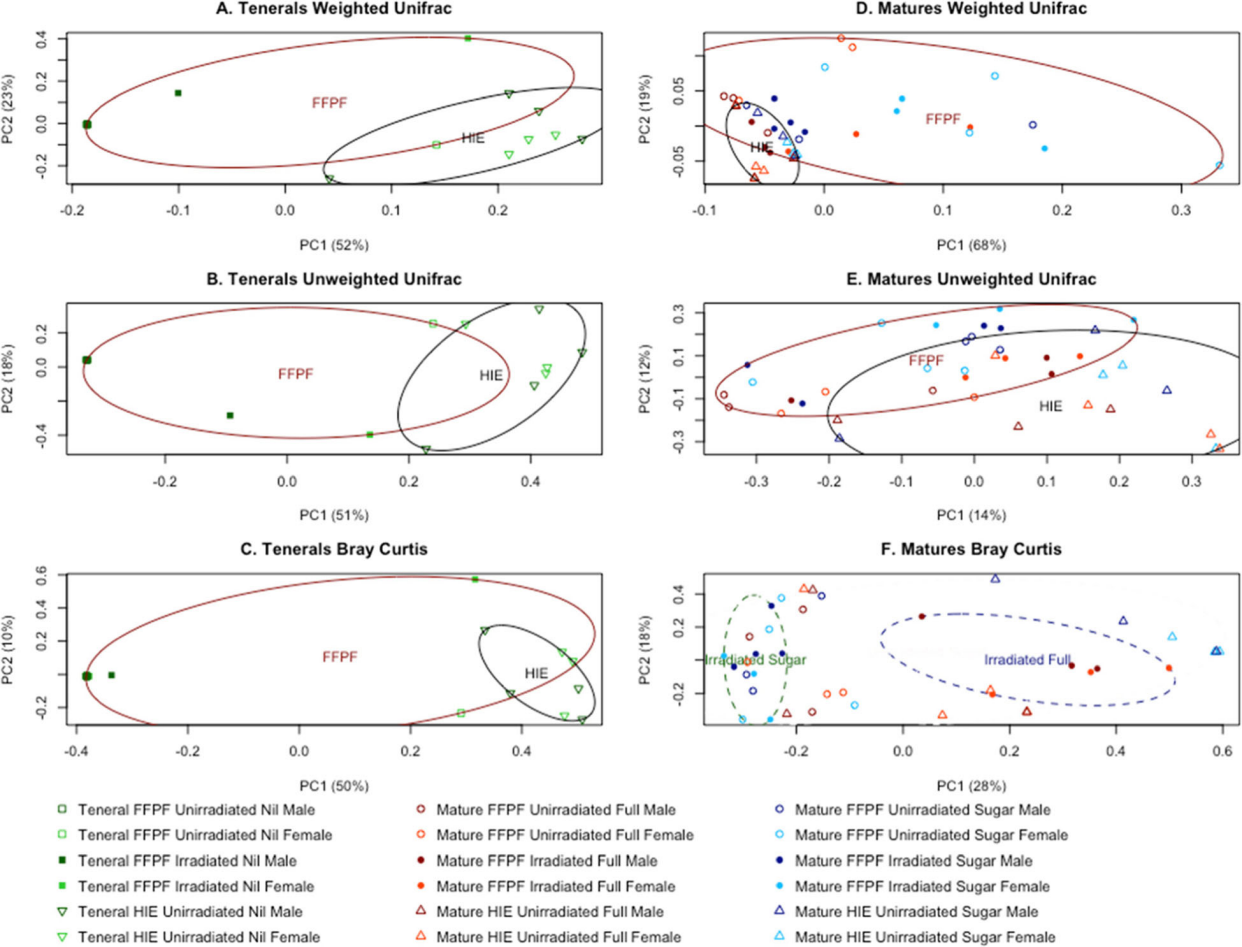
Principal coordinate analysis of (**a**) weighted UniFrac distances of tenerals, (**b**) unweighted UniFrac distances of tenerals, (**c**) Bray-Curtis distances of tenerals, (**d**) weighted UniFrac distances of matures, (**e**) unweighted UniFrac distances of matures, and (**f**) Bray-Curtis distances of matures

Beta diversity measurements were applied to sequences clustered at 97% similarity using the weighted and unweighted UniFrac and Bray-Curtis distances (Fig. 4). In the tenerals, the PCoA of all three measurements indicated an emerging pattern of separation between samples based on colony origin. This pattern was also visible in mature flies. Further to this, the Bray-Curtis distance PCoA within the irradiated mature flies showed a separation between flies fed a full adult diet, and those fed an adult diet of sugar only. No sex effect was observed in the PCoAs.

### Identity of dominant bacterial OTUs

For the entire dataset, the nine most abundant OTUs represented over 80% of the rarefied combined mature and teneral sequence reads (Table 2, Additional file 3). Based on the BLAST search of the short 16S rRNA gene amplicons, these dominant OTUs likely belonged to the Enterobacteriaceae genera *Enterobacter* (1 OTU), *Pluralibacter/Klebsiella* (2 OTUs)*, Proteus* (1 OTU), *Providencia* (2 OTUs) and *Serratia* (2 OTUs), and to the Acetobacteraceae genus *Asaia* (1 OTU).

**Table 2:** Major OTUs (excluding OTUs less than 1%) in teneral and mature adult *Bactrocera tryoni* and their BLAST hits

OTU ID	Tenerals		Mature		Combine		Query cover (%)	E value	Identity score (%)	Closest NCBI BLAST hit
	Reads	Abundance	Reads	Abundance	Reads	Abundance				
4,418,165	5	2.63%	44,809	20.89%	44,814	20.87%	100%	0.0	99%	*Pluralibacter gergoviae* strain BYK-7 16S rRNA gene, complete sequence; *Pluralibacter gergoviae* strain FB2, complete genome; *Klebsiella oxytoca* strain CAV1015, complete genome; *Klebsiella oxytoca* strain CAV1099, complete genome
1,122,622	8	4.21%	35,835	16.71%	35,843	16.70%	100%	0.0	100%	*Providencia rettgeri* strain RB151, complete genome
3,101,394	6	3.16%	33,454	15.60%	33,460	15.59%	100%	0.0	100%	*Providencia rettgeri* strain RB151, complete genome
470,879	7	3.68%	32,797	15.29%	32,804	15.28%	100%	0.0	100%	*Proteus* sp. strain JP20 16S rRNA gene, partial sequence
814,266	97	51.05%	25,055	11.68%	25,152	11.72%	100%	0.0	100%	*Asaia bogorensis* NBRC 16594 DNA, complete genome
4,477,719	1	0.53%	10,079	4.70%	10,080	4.70%	100%	0.0	99%	*Pluralibacter gergoviae* strain BYK-7 16S rRNA gene, complete sequence; *Pluralibacter gergoviae* strain FB2, complete genome; *Klebsiella oxytoca* strain CAV1015, complete genome; *Klebsiella oxytoca* strain CAV1099, complete genome
1,108,706	2	1.05%	9058	4.22%	9060	4.22%	100%	0.0	100%	*Serratia marcescens* strain B3R3, complete genome
572,750	0	0.00%	7304	3.41%	7304	3.40%	100%	0.0	100%	*Enterobacter* sp. Amlc14 16S rRNA gene, partial sequence
4,343,005	0	0.00%	6554	3.06%	6554	3.05%	100%	0.0	100%	*Serratia marcescens* strain B3R3, complete genome

**Fig. 5:**
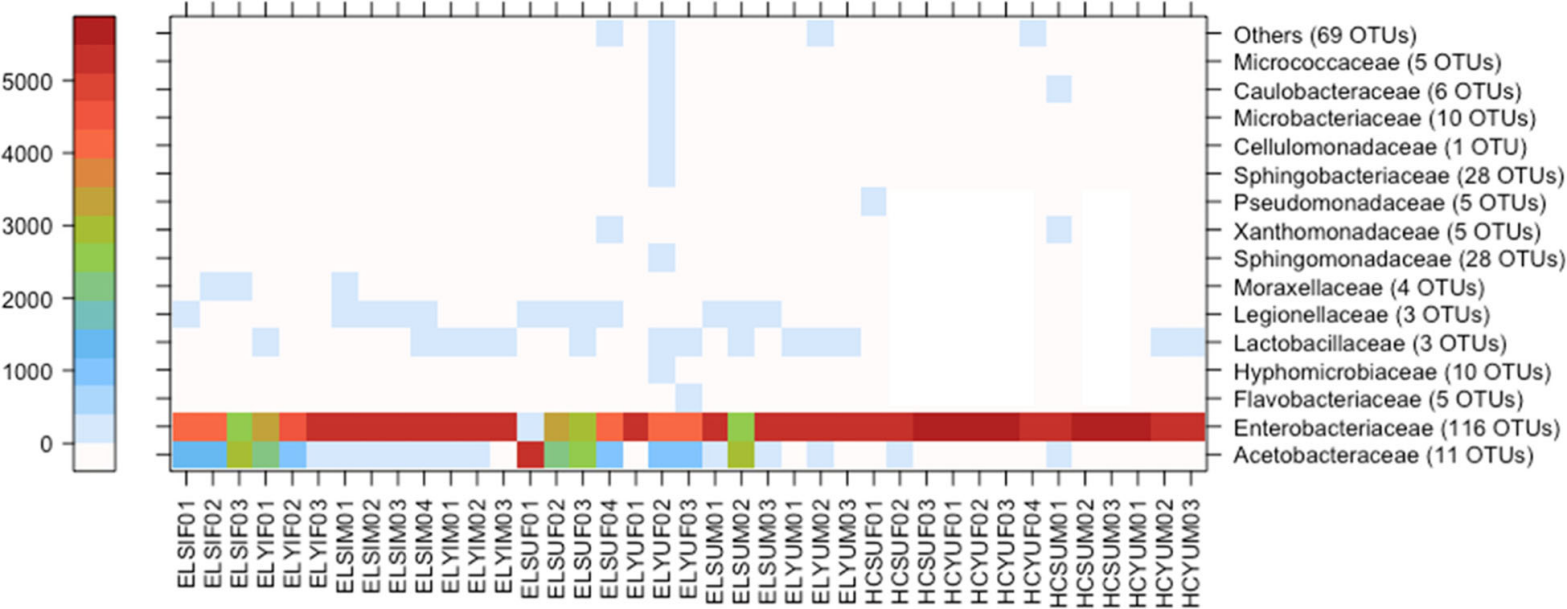
Relationship between individual mature *Bactrocera tryoni* and major bacterial families. Sample name letter codes are as per Table 1

In mature flies, the most abundant and OTU diverse bacterial family was Enterobacteriaceae, comprising 116 OTUs (Fig. 5). The high abundance of Enterobacteriaceae in matures was mostly due to 8 OTUs that accounted for over 86% of the total rarefied mature adult sequence reads (Table 2). The second most abundant bacterial family in the mature flies was Acetobacteraceae, where one of the 11 OTUs accounted for 11% of the total rarefied mature adult sequence reads. Based on a BLAST search, this dominant Acetobacteraceae OTU belonged to the genus *Asaia*.

**Fig. 6:**
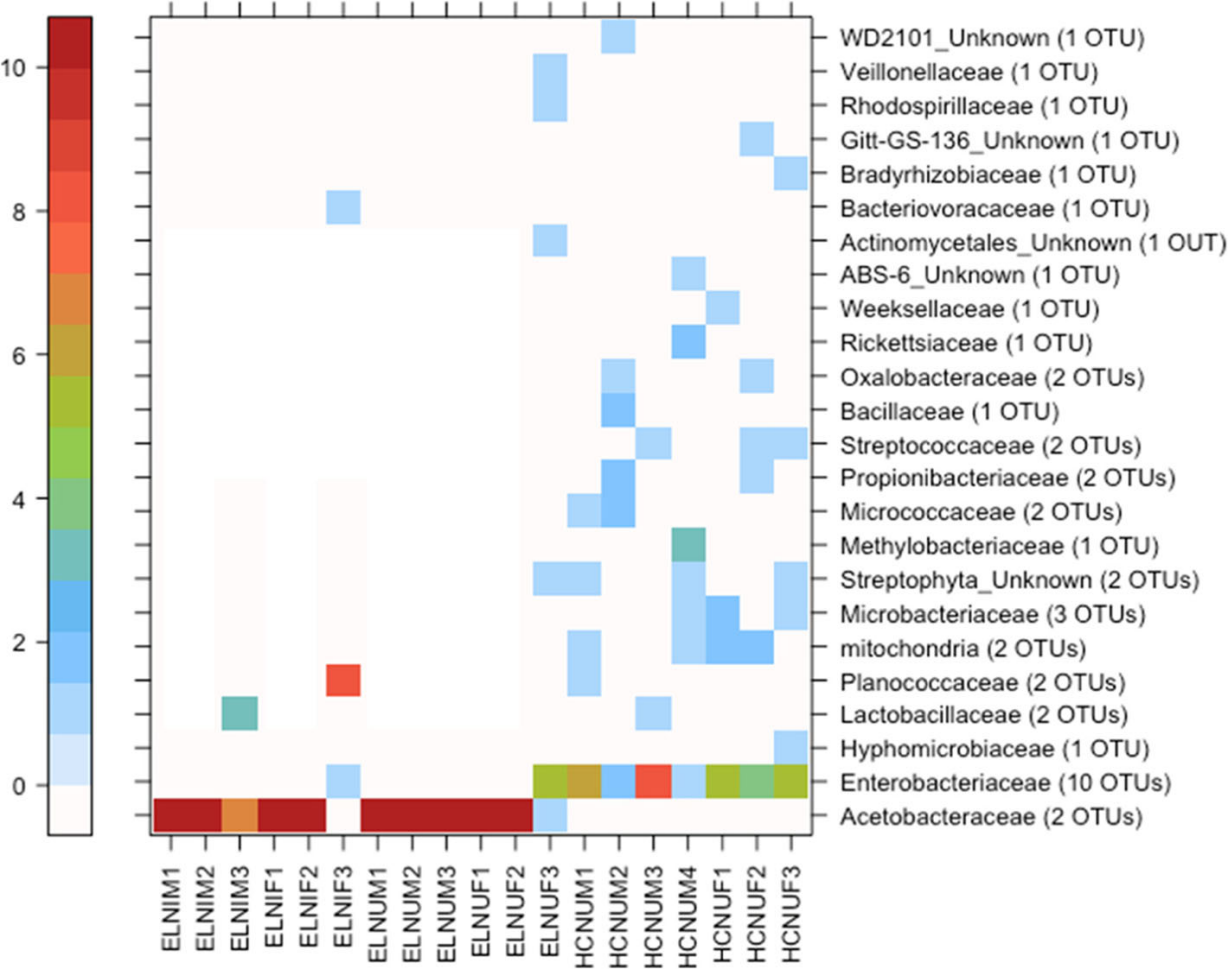
Relationship between individual teneral *Bactrocera tryoni* and bacterial families. Sample name letter codes are as per Table 1

The HIE tenerals harboured 10 OTUs classified to Enterobacteriaceae, and were dominated by the same Enterobacteriaceae OTUs that were highly abundant in the mature flies (Table 2). The FFPF tenerals contained two Acetobacteraceae OTUs, but were dominated by one OTU that accounted for 51% of the total rarefied teneral sequence reads (Fig. 6). The dominant Acetobacteraceae OTU in tenerals, as in matures, was *Asaia*. Other notable OTUs in tenerals included Planococcaceae (according to the short 16S rRNA gene amplicon possibly a *Staphylococcus* sp.), and mitochondrial 16S rRNA gene from Poaceae (grasses), probably from the cane sugar used in the FFPF larval diet, and hits to a chloroplast 16S rRNA gene. *Asaia* or other Acetobacteraceae were not found in teneral and mature HIE flies.

**Fig. 7:**
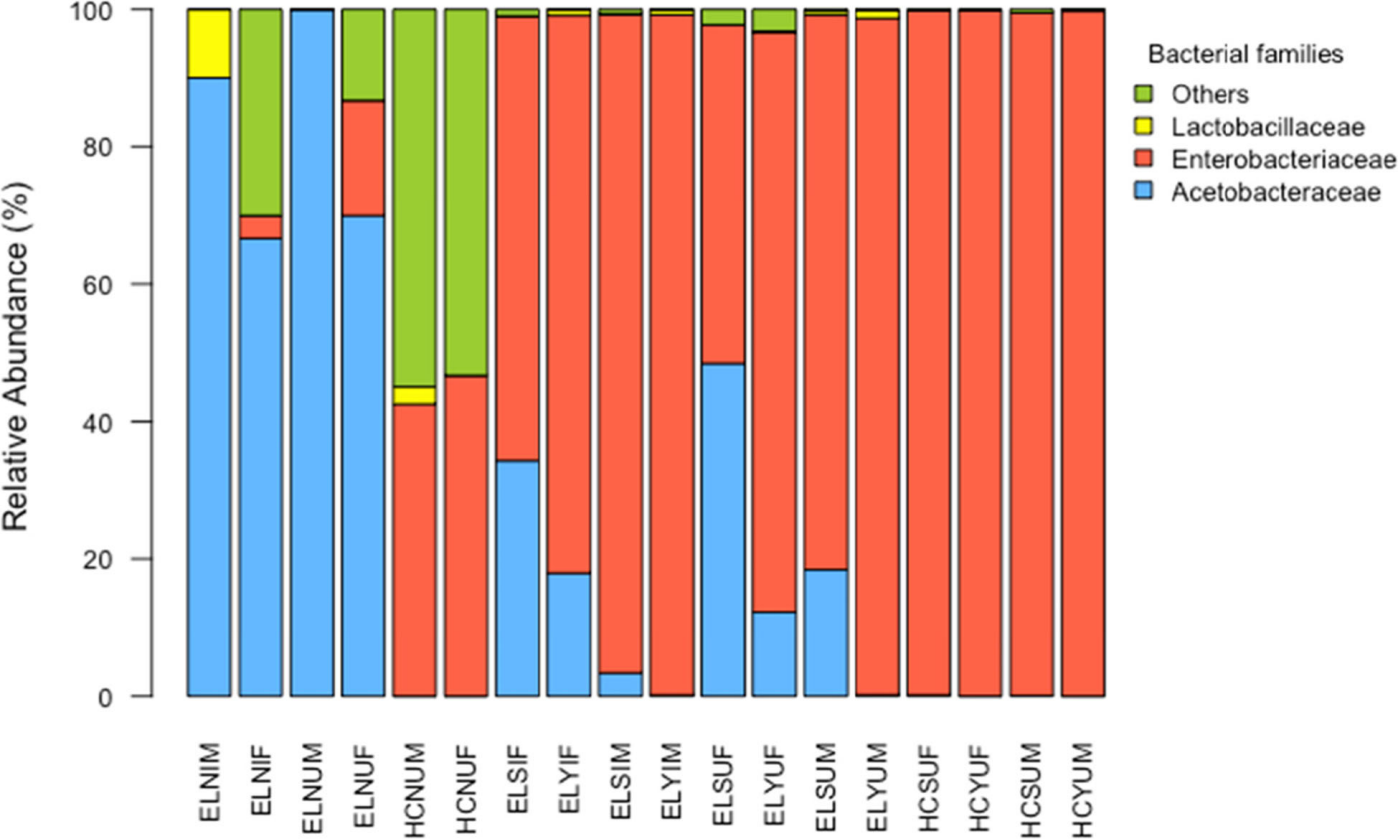
Relative abundance of bacterial families in teneral and mature *Bactrocera tryoni* treatment groups represented by 16S rRNA gene sequences after rarefaction of tenerals to 10 sequence reads and mature adults to 5500 sequence reads. Letter codes are as per Table 1

The relative abundance plot (Fig. 7) suggested a pattern in mature FFPF flies (which were dominated by Acetobacteraceae in the teneral stage) that favoured, irrespective of irradiation, the proliferation of Enterobacteriaceae (and reduced relative presence of Acetobacteraceae) when fed the full adult diet over those fed the sugar only adult diet. Furthermore, for FFPF matures, it appeared that females had higher relative abundance of Acetobacteraceae than males when fed sugar however this was not observed when FFPF flies were fed a full adult diet.

## Discussion

We used 16S rRNA gene amplicon sequencing to characterise the bacterial community composition and structure of individual adult *B. tryoni* and to evaluate the impact of colony origin, adult diets and irradiation on the bacterial community across two adult developmental stages. Tenerals consistently had reduced total bacterial titres when compared with mature adult flies. This may be due to the bottleneck that bacterial populations experience as a consequence of the emptying of gut content prior to pupation during holometabolous metamorphosis. An overall low bacterial count in larvae, pupae and teneral flies was also observed for Mediterranean fruit fly, *Ceratitis capitata* (Wiedemann)*,* (Diptera: Tephritidae) [31]. Another possible explanation for the differences found between teneral and mature flies may be that larvae are less mobile and restricted to one diet while pupae are a non-feeding, largely sessile stage and, therefore, have reduced exposure to diverse microbial communities compared with adults. Due to their mobility, adult flies have the potential to forage from diverse food sources across environments with variable microbial communities (particularly in the field and to some extent when in a captive colony).

Unexpectedly, irradiated mature *B. tryoni* had a higher bacterial sequence read volume than unirradiated individuals, suggesting an increased bacterial load. Given that such irradiation effects on gut bacterial communities have not yet been investigated in other tephritids, our findings warrant further investigation of bacterial population dynamics in irradiated flies. Despite the damage caused by irradiation on a tephritid gut [13], previous research demonstrated that irradiated tephritids still display normal proteolytic activity [32]. Therefore, the damage caused by irradiation may enable some bacteria to exploit newly available resources, and/or compensate for the damage. Alternatively, we can also postulate that the damage caused by irradiation allows some bacteria to proliferate in the gut due to an inability of irradiated fruit flies to regulate their bacterial load. However, this increased bacterial load did not impact the total bacterial diversity and relative abundance of OTUs.

Although the effects of adult diets on tephritids have been well characterised [33–35], to date little is known about the impact of diets on their microbiome [36]. The comparison of OTU diversity between teneral and mature adults revealed a clear distinction between flies with different colony origins (at FFPF and HIE, flies were reared on different larval diets in different environments and were sourced from different field populations in different years). This is consistent with the PCoA plots (Fig. 4 a, b, c, d, and e) where an emerging separation was visible between samples from different colony origins.

The colony origin significantly influenced the number of sequence reads in tenerals and the number of OTUs in both tenerals and matures. The flies from the FFPF and HIE were derived from different lines. Further, the FFPF line had been captive for under 2 years, while the HIE colony had been established for 6 years. Fruit flies are known to lose their field characteristics in as little as three generations [5, 37] as they become domesticated. Further to this, FFPF flies were reared at higher densities compared to the HIE flies and this may have impacted the stress of the environment for developing larvae, and prior generations of adult flies. Therefore, different host genotypes and environmental influences such as larval densities could play a part in the different bacterial community composition observed between flies originating from the FFPF and HIE. Although we cannot entirely separate the effects of larval diet, larval environment (including density of larvae in the diet) and domestication history of the two fly colonies, it remains likely that the different bulking agents used in the larval diets of FFPF and HIE were probably important contributors to the observed differences in the bacterial community in tenerals. Regardless of the pupal origin, as the adult flies matured within the same environment, the bacterial communities became increasingly similar; therefore, the adult environment impacted the bacterial communities of flies as they developed.

Besides this effect of colony origin, our study of captive *B. tryoni* indicates that, within diet treatments, the bacterial communities were similar in composition between male and female flies. Therefore, we can exclude any sex effects on bacterial community composition in captive flies.

Based on the short 16S rRNA gene amplicons, the genera of the dominant OTUs in the reared populations for *B. tryoni* were *Enterobacter*, *Pluralibacter*/*Klebsiella*, *Proteus*, *Providencia* and *Serratia* (Enterobacteriaceae) and *Asaia* (Acetobacteraceae). The dominance by Enterobacteriaceae supports previous findings from microbiome studies of *B. tryoni* [11, 38], and other tephritids of the *Bactrocera* genus including *B. cacuminata* [11, 38], *B. carambolae* [39], *B. cucurbitae* [40, 41], *B. dorsalis* [39, 42–45], *B. jarvisi* [11], *B. neohumeralis* [11], *B. minax* [46], *B. oleae* [47, 48], *B. tau* [49–51] and *B. zonata* [52].

Teneral *B. tryoni* originating from FFPF pupae were dominated by Acetobacteraceae (mostly *Asaia*) but, in the mature stage, these flies had a lower proportional representation of this bacterial family than Enterobacteriaceae, and provision of a full adult diet exacerbated this effect. This may suggest that the ratio of carbohydrates and proteins in the adult diet may shift bacterial community structure. Nitrogen, the key element in proteins, is considered to be a limiting factor in the reproductive success of both male and female *C. capitata* [53, 54]. Despite the provision of yeast as a protein source, nitrogen is paradoxically limited [55]. Enterobacteriaceae are known to contain diazotrophic species [56] which would assist in providing more or specifically required forms of nitrogen. This would explain the abundance of Enterobacteriaceae in mature adult *B. tryoni*. Enterobacteriaceae species have also been credited for improving egg production in female *B. oleae* [15, 47] and improved mating performance in male *C. capitata* [12, 57]. These studies have sparked the research interest into the use of Enterobacteriaceae candidates to enhance performance of *B. tryoni* [58, 59].

The high abundance of *Asaia* in *B. tryoni* adult flies reared from FFPF pupae (but not seen in adult HIE flies) is a novel finding as previous studies found *Asaia* only at low abundance in adult *B. tryoni* [11] and *B. oleae* [60]. Furthermore, *Asaia* has recently been detected at high abundance in *B. tryoni* larvae obtained from field collected fruit, and in larvae reared in artificial diets [61]. The role of *Asaia* sp. in tephritids is still unknown, however, bacteria of this genus are dominant taxa in the microbiota of larvae and several adult mosquitoes (*Anopheles gambiae*, *A. maculipennis* and *A. stephensi)* [62, 63]. *Asaia* spp. have been found to be important in the development of *A. stephensi* as when deprived of it, larval development was delayed [64].

## Conclusion

Our study has shown that the microbiome of *B. tryoni* during adult development is impacted by irradiation, the environment and the adult diet, with a very similar microbiome shared between male and female captive and domesticated *B. tryoni*. Symbiotic bacteria have previously been supplemented to larval and adult diets of other tephritid pest species with the aim to improve the performance of mass-reared flies in SIT programmes [2]. Our findings demonstrate that colony origin (in our study, compounded by differences in larval diets, rearing environments, field source populations and duration of domestication) and adult diets impact mature *B. tryoni* gut microbiota. However, diet composition (such as the ratio of carbohydrates and protein) is evidently an important factor for the application of fruit fly probiotics. Importantly, our work also suggests that the ideal time to introduce a probiotic to impact the mature adult tephritids’ microbiota is from the teneral stage, which is consistent with the 2 to 3 day pre-release holding period for sterile adult *B. tryoni* during which the adults are provided with food and water [65]. The dominance of the bacterial families Enterobacteriaceae and Acetobacteraceae, specifically *Asaia* sp., warrants more research into the association of these bacteria with *B. tryoni*, particularly in understanding the role they currently play in mass-rearing and performance of the sterile individuals released in SIT programmes.

## Supplementary information

**Additional file 1: Table S1.** Alpha diversity metrics of 58 gut samples from *Bactrocera tryoni* reared on artificial diets, calculated at 97% identity level, after rarefaction of tenerals to 10 sequence reads and matures to 5500 sequence reads. **Table S2.** ANOVA of 16S rRNA gene sequence reads of teneral and mature adult *Bactrocera tryoni.* **Table S3.** ANOVA of number of OTUs in *Bactrocera tryoni*.

**Additional file 2. **Representative set of 16S rRNA gene sequences of OTUs from teneral and mature *Bactrocera tryoni.*

**Additional file 3: Table S4.** OTU Table.

## Data Availability

The datasets generated and/or analysed during the current study are available as attachment in the Additional files and also in the NCBI SRA repository, BioProject ID: PRJNA579218.

## Abbreviations

ANSTO: Australian Nuclear Science and Technology Organisation
CCPIC: Central Coast Primary Industries Centre
DPI: Department of Primary Industries
FFPF: Fruit Fly Production Facility
HIE: Hawkesbury Institute for the Environment
NSW: New South Wales
NGS: Next Generation Sequencing
OTU: Operational Taxonomic Unit
PCoA: Principal Component Analysis
SIT: Sterile Insect Technique
