# Supplementary material for: Diet and irradiation effects on the bacterial community composition and structure in the gut of domesticated teneral and mature Queensland fruit fly, Bactrocera tryoni (Diptera: Tephritidae)
Source: BMC Microbiol. 2019 Dec 24;19(Suppl 1):281. doi: 10.1186/s12866-019-1649-6 (PMC6929413; doi:10.1186/s12866-019-1649-6)
Supplement: Supplementary file 1 — Additional file 1: Table S1. Alpha diversity metrics of 58 gut samples from Bactrocera tryoni reared on artificial diets, calculated at 97% identity level, after rarefaction of tenerals to 10 sequence reads and matures to 5500 sequence reads. Table S2. ANOVA of 16S rRNA gene sequence reads of teneral and mature adult Bactrocera tryoni. Table S3. ANOVA of number of OTUs in Bactrocera tryoni. [file 12866_2019_1649_MOESM1_ESM.docx]

Additional File 1

**Additional Table S1:** Alpha diversity metrics of 58 gut samples from *Bactrocera tryoni* reared on artificial diets, calculated at 97% identity level, after rarefaction of tenerals to 10 sequence reads and matures to 5,500 sequence reads.

| Sample ID | Raw sequence reads | Sequences after OTU picking and singleton removal | Sequences after chimera removal | OTUs before rarefaction | OTUs after rarefaction | Chao1 | Simpson | Shannon | Good |
| --- | --- | --- | --- | --- | --- | --- | --- | --- | --- |
| ELNIF01 | 36,136 | 19,658 | 19,606 | 25 | 2 | 2 | 0.18 | 0.469 | 0.9 |
| ELNIF02 | 17,530 | 7,943 | 7,911 | 17 | 1 | 1 | 0 | 0 | 1 |
| ELNIF03 | 1,223 | 118 | 88 | 19 | 3 | 4 | 0.34 | 0.922 | 0.8 |
| ELNIM01 | 1,461 | 173 | 169 | 12 | 1 | 1 | 0 | 0 | 1 |
| ELNIM02 | 858 | 166 | 156 | 14 | 1 | 1 | 0 | 0 | 1 |
| ELNIM03 | 2,794 | 1,239 | 1,228 | 14 | 2 | 2 | 0.42 | 0.881 | 1 |
| ELNUF01 | 1,662 | 704 | 695 | 10 | 1 | 1 | 0 | 0 | 1 |
| ELNUF02 | 2,264 | 1,001 | 990 | 5 | 1 | 1 | 0 | 0 | 1 |
| ELNUF03 | 657 | 17 | 15 | 8 | 8 | 29 | 0.84 | 2.846 | 0.3 |
| ELNUM01 | 1,315 | 457 | 455 | 12 | 1 | 1 | 0 | 0 | 1 |
| ELNUM02 | 8,089 | 3,789 | 3,776 | 15 | 1 | 1 | 0 | 0 | 1 |
| ELNUM03 | 2,374 | 1,084 | 1,071 | 16 | 1 | 1 | 0 | 0 | 1 |
| HCNUF01 | 1,369 | 42 | 28 | 13 | 7 | 8.5 | 0.84 | 2.722 | 0.6 |
| HCNUF02 | 511 | 37 | 24 | 12 | 9 | 23 | 0.88 | 3.122 | 0.2 |
| HCNUF03 | 365 | 37 | 24 | 15 | 10 | 55 | 0.9 | 3.322 | 0 |
| HCNUM01 | 511 | 17 | 11 | 8 | 8 | 13 | 0.86 | 2.922 | 0.4 |
| HCNUM02 | 383 | 38 | 15 | 9 | 7 | 8.5 | 0.84 | 2.722 | 0.6 |
| HCNUM03 | 2,575 | 46 | 41 | 16 | 6 | 9 | 0.76 | 2.322 | 0.6 |
| HCNUM04 | 822 | 20 | 12 | 7 | 7 | 12 | 0.82 | 2.646 | 0.5 |
| ELSIF01 | 79,340 | 36,085 | 32,718 | 56 | 31 | 40 | 0.814 | 2.742 | 1 |
| ELSIF02 | 58,176 | 26,221 | 25,563 | 52 | 35 | 57.75 | 0.76 | 2.403 | 1 |
| ELSIF03 | 119,749 | 61,442 | 57,800 | 69 | 32 | 51.5 | 0.634 | 1.91 | 1 |
| ELYIF01 | 35,187 | 16,531 | 15,873 | 34 | 23 | 29 | 0.758 | 2.352 | 1 |
| ELYIF02 | 66,777 | 27,562 | 26,322 | 49 | 34 | 52.2 | 0.733 | 2.35 | 1 |
| ELYIF03 | 46,307 | 20,385 | 18,918 | 35 | 23 | 28.25 | 0.635 | 1.858 | 1 |
| ELSIM01 | 54,123 | 23,648 | 20,820 | 58 | 43 | 78 | 0.605 | 2.149 | 1 |
| ELSIM02 | 135,635 | 53,193 | 46,204 | 63 | 25 | 30 | 0.508 | 1.558 | 1 |
| ELSIM03 | 116,225 | 50,035 | 41,522 | 70 | 34 | 43.333 | 0.734 | 2.445 | 1 |
| ELSIM04 | 32,740 | 14,293 | 12,569 | 50 | 39 | 66.5 | 0.743 | 2.389 | 1 |
| ELYIM01 | 98,640 | 44,663 | 42,832 | 89 | 43 | 100 | 0.778 | 2.551 | 1 |
| ELYIM02 | 72,273 | 31,285 | 29,982 | 63 | 35 | 44 | 0.734 | 2.361 | 1 |
| ELYIM03 | 92,122 | 39,619 | 37,117 | 71 | 37 | 64.5 | 0.73 | 2.404 | 1 |
| ELSUM01 | 55,218 | 31,779 | 31,328 | 38 | 19 | 20 | 0.111 | 0.474 | 1 |
| ELSUM02 | 53,557 | 25,441 | 22,502 | 57 | 33 | 57 | 0.536 | 1.38 | 1 |
| ELSUM03 | 47,330 | 23,637 | 22,594 | 43 | 27 | 34.2 | 0.603 | 1.674 | 1 |
| ELSUM04 | 36,958 | 16,781 | 13,822 | 45 | 35 | 65.6 | 0.546 | 1.681 | 1 |
| ELYUF01 | 38,547 | 15,332 | 14,565 | 50 | 40 | 67.2 | 0.789 | 2.548 | 1 |
| ELYUF02 | 46,673 | 21,248 | 20,529 | 157 | 102 | 159.652 | 0.695 | 2.215 | 0.99 |
| ELYUF03 | 68,238 | 32,431 | 30,788 | 52 | 27 | 29.5 | 0.702 | 2.118 | 1 |
| ELSUM01 | 58,925 | 23,937 | 22,739 | 40 | 27 | 40.75 | 0.39 | 1.154 | 1 |
| ELSUM02 | 43,897 | 20,943 | 19,648 | 49 | 29 | 42.75 | 0.652 | 1.944 | 1 |
| ELSUM03 | 45,760 | 19,573 | 18,803 | 40 | 19 | 20.5 | 0.465 | 1.439 | 1 |
| ELYUM01 | 34,237 | 14,272 | 14,084 | 55 | 41 | 48.091 | 0.792 | 2.587 | 1 |
| ELYUM02 | 76,272 | 34,002 | 31,888 | 72 | 44 | 54.909 | 0.745 | 2.471 | 1 |
| ELYUM03 | 62,358 | 28,154 | 27,490 | 77 | 43 | 120 | 0.756 | 2.499 | 1 |
| HCSUF01 | 34,712 | 15,292 | 14,755 | 30 | 20 | 29.333 | 0.126 | 0.462 | 1 |
| HCSUF02 | 39,241 | 16,652 | 16,564 | 24 | 17 | 20.333 | 0.082 | 0.354 | 1 |
| HCSUF03 | 65,188 | 27,074 | 26,996 | 44 | 16 | 17 | 0.365 | 1.144 | 1 |
| HCYUF01 | 76,875 | 34,783 | 32,586 | 36 | 21 | 24.75 | 0.236 | 0.76 | 1 |
| HCYUF02 | 60,806 | 26,958 | 25,065 | 25 | 12 | 17 | 0.009 | 0.056 | 1 |
| HCYUF03 | 72,492 | 32,319 | 31,318 | 40 | 21 | 23 | 0.385 | 1.195 | 1 |
| HCYUF04 | 31,498 | 14,232 | 7,850 | 26 | 22 | 88 | 0.365 | 1.099 | 1 |
| HCSUM01 | 32,941 | 14,502 | 14,176 | 38 | 26 | 44.333 | 0.615 | 1.766 | 1 |
| HCSUM02 | 60,550 | 26,857 | 25,465 | 34 | 16 | 19.75 | 0.058 | 0.268 | 1 |
| HCSUM03 | 44,810 | 20,220 | 19,856 | 34 | 27 | 40.2 | 0.469 | 1.428 | 1 |
| HCYUM01 | 62,267 | 25,528 | 24,839 | 32 | 20 | 29.333 | 0.374 | 1.122 | 1 |
| HCYUM02 | 76,692 | 32,602 | 31,923 | 35 | 19 | 34 | 0.113 | 0.406 | 1 |
| HCYUM03 | 37,451 | 12,386 | 12,011 | 22 | 15 | 24.333 | 0.019 | 0.107 | 1 |

Sample IDs are as per Table 1. OTUs = number of operational taxonomic units calculated at 97 % similarity; Chao1= estimate of species richness; Shannon and Simpson indices estimate diversity; Good’s equation measures coverage.

Additional Table 2: ANOVA of 16S rRNA gene sequence reads of teneral and mature adult *Bactrocera tryoni.*

| **Treatment factors** | | **All** | | | | | **Tenerals** | | | | | **Mature** | | | | |
| --- | --- | --- | --- | --- | --- | --- | --- | --- | --- | --- | --- | --- | --- | --- | --- | --- |
|  |  | n | *x̅* | SE | *F* | *p* | n | *x̅* | SE | *F* | *p* | n | *x̅* | SE | *F* | *p* |
| Life stage | Tenerals | 19 | 1911.316 | 1076.351 | 85.149 | <0.001*** |  |  |  |  |  |  |  |  |  |  |
|  | Mature | 39 | 25190.359 | 1674.838 |  |  |  |  |  |  |  |  |  |  |  |  |
| Irradiation (FFPF) | Irradiated | 19 | 23020.950 | 3939.679 | 2.412 | 0.129 | 6 | 4859.667 | 3198.175 | 1.296 | 0.282 | 13 | 31403.08 | 3676.836 | 4.8921 | 0.03675* |
|  | Unirradiated | 19 | 15672.740 | 2620.421 |  |  | 6 | 1167.000 | 544.801 |  |  | 13 | 22367.69 | 1780.026 |  |  |
| Sex | Male | 29 | 17272.480 | 2758.424 | 0.024 | 0.877 | 9 | 693.400 | 370.694 | 1.459 | 0.244 | 19 | 25998.32 | 2400.5 | 0.2165 | 0.6444 |
|  | Female | 29 | 17856.450 | 2553.224 |  |  | 10 | 3264.556 | 2212.767 |  |  | 20 | 24422.8 | 2385.374 |  |  |
| Colony origin (Unirradiated) | FFPF | 19 | 15672.740 | 2620.421 | 0.152 | 0.699 | 6 | 1167.000 | 544.801 | 5.231 | 0.043* | 13 | 22367.69 | 1780.026 | 0.0388 | 0.8454 |
|  | HIE | 20 | 14177.950 | 2789.607 |  |  | 7 | 22.143 | 4.008 |  |  | 13 | 21800.31 | 2263.295 |  |  |
| Adult diet (Mature) | Full |  |  |  |  |  |  |  |  |  |  | 19 | 25051.58 | 2132.357 | 0.0064 | 0.9369 |
|  | Sugar |  |  |  |  |  |  |  |  |  |  | 20 | 25322.2 | 2616.427 |  |  |

Significance codes: ‘***’ 0.001 ‘**’ 0.01 ‘*’ 0.05 ‘.’ 0.1 ‘ ’ 1

n = sample size, *x̅* = mean, SE = standard error, *F* = F value, *p* = P value

Additional Table 3: ANOVA of number of OTUs in *Bactrocera tryoni.*

| **Treatment factors** | | **All** | | | | | **Tenerals** | | | | | **Mature** | | | | |
| --- | --- | --- | --- | --- | --- | --- | --- | --- | --- | --- | --- | --- | --- | --- | --- | --- |
|  |  | n | *x̅* | SE | *F* | *p* | n | *x̅* | SE | *F* | *p* | n | *x̅* | SE | *F* | *p* |
| Life stage | Tenerals | 19 | 4.053 | 0.774 | 55.979 | <0.001*** |  |  |  |  |  |  |  |  |  |  |
|  | Mature | 39 | 30.051 | 2.386 |  |  |  |  |  |  |  |  |  |  |  |  |
| Irradiation (FFPF) | Irradiated | 19 | 23.368 | 3.692 | 0.189 | 0.666 | 6 | 1.667 | 0.333 | 0.170 | 0.689 | 13 | 33.385 | 1.842 | 0.424 | 0.521 |
|  | Unirradiated | 19 | 26.263 | 5.540 |  |  | 6 | 2.167 | 1.167 |  |  | 13 | 37.385 | 5.864 |  |  |
| Sex | Male | 29 | 21.276 | 2.876 | 0.013 | 0.911 | 10 | 3.500 | 0.969 | 0.552 | 0.468 | 19 | 30.632 | 2.301 | 0.055 | 0.816 |
|  | Female | 29 | 21.793 | 3.605 |  |  | 9 | 4.667 | 1.258 |  |  | 20 | 29.500 | 4.171 |  |  |
| Colony origin (Unirradiated) | FFPF | 19 | 26.263 | 5.540 | 3.819 | 0.058. | 6 | 2.167 | 1.167 | 20.928 | <0.001*** | 13 | 37.385 | 5.864 | 9.055 | 0.006** |
|  | HIE | 20 | 15.300 | 1.494 |  |  | 7 | 7.714 | 0.522 |  |  | 13 | 19.385 | 1.180 |  |  |
| Adult diet (Mature) | Full |  |  |  |  |  |  |  |  |  |  | 19 | 32.737 | 4.521 | 1.211 | 0.278 |
|  | Sugar |  |  |  |  |  |  |  |  |  |  | 20 | 27.500 | 1.760 |  |  |

Significance codes: ‘***’ 0.001 ‘**’ 0.01 ‘*’ 0.05 ‘.’ 0.1 ‘ ’ 1

n = sample size, *x̅* = mean, SE = standard error, *F* = F value, *p* = P value
